# Association of Central Sensitization Inventory Scores With Pain Outcomes After Endometriosis Surgery

**DOI:** 10.1001/jamanetworkopen.2023.0780

**Published:** 2023-02-27

**Authors:** Natasha L. Orr, Alice J. Huang, Yang Doris Liu, Heather Noga, Mohamed A. Bedaiwy, Christina Williams, Catherine Allaire, Paul J. Yong

**Affiliations:** 1 Department of Obstetrics and Gynecology, University of British Columbia, Vancouver, British Columbia, Canada; 2 University of British Columbia Endometriosis and Pelvic Pain Laboratory, Vancouver, British Columbia, Canada; 3 Women’s Health Research Institute, Vancouver, British Columbia, Canada

## Abstract

**Importance:**

A subset of people who undergo surgery for endometriosis have persistent pain, suggesting that other factors besides the endometriosis, such as central sensitization, may play a role in this pain. The Central Sensitization Inventory, a validated self-reported questionnaire of central sensitization symptoms, may identify individuals with endometriosis who have more pain after surgery due to pain sensitization.

**Objective:**

To examine whether greater baseline Central Sensitization Inventory scores are associated with postsurgical pain outcomes.

**Design, Setting, and Participants:**

This prospective, longitudinal cohort study performed at a tertiary center for endometriosis and pelvic pain in British Columbia, Canada, included all patients aged 18 to 50 years with diagnosed or suspected endometriosis and a baseline visit between January 1, 2018, and December 31, 2019, who underwent surgery after the baseline visit. Individuals who were menopausal, had a prior hysterectomy, or were missing data for outcomes or measures were excluded. Data analysis was performed from July 2021 to June 2022.

**Main Outcomes and Measures:**

The primary outcome was chronic pelvic pain at follow-up measured on a scale of 0 to 10, with 0 to 3 indicating no pain or mild pain, 4 to 6 indicating moderate pain, and 7 to 10 indicating severe pain. Secondary outcomes were deep dyspareunia, dysmenorrhea, dyschezia, and back pain at follow-up. The main variable of interest was baseline Central Sensitization Inventory score (measured from 0 to 100, consisting of 25 self-reported questions rated from 0 to 4 [never, rarely, sometimes, often, and always, respectively]).

**Results:**

A total of 239 patients (mean [SD] age, 34 [7] years; 189 [79.1%] White [11 (5.8%) identified as White mixed with another ethnicity], 1 [0.4%] Black or African American, 29 [12.1%] Asian, 2 [0.8%] Native Hawaiian or Pacific Islander, 16 [6.7%] other, and 2 [0.8%] mixed race or ethnicity) with follow-up data at more than 4 months after surgery were included in this study (71.0% follow-up rate). The mean (SD) baseline Central Sensitization Inventory score was 43.8 (18.2), and the mean (SD) follow-up was 16.1 (6.1) months. Higher baseline Central Sensitization Inventory scores were significantly associated with higher chronic pelvic pain (odds ratio [OR], 1.02; 95% CI, 1.00-1.03; *P* = .02), deep dyspareunia (OR, 1.03; 95% CI, 1.01-1.04; *P* = .004), dyschezia (OR, 1.03; 95% CI, 1.01-1.04; *P* < .001), and back pain (OR, 1.02; 95% CI, 1.00-1.03; *P* = .02) at follow-up, when controlling for baseline pain scores. The Central Sensitization Inventory scores themselves decreased slightly from baseline to follow-up (mean [SD] score, 43.8 [18.2] vs 41.7 [18.9]; *P* = .05); however, individuals with high baseline Central Sensitization Inventory scores still had high scores at follow-up.

**Conclusions and Relevance:**

In this cohort study of 239 patients with endometriosis, higher Central Sensitization Inventory scores at baseline were associated with worse pain outcomes after endometriosis surgery, when controlling for baseline pain scores. The Central Sensitization Inventory could be used to counsel patients with endometriosis on their expected outcomes after surgery.

## Key Points

**Question:**

Are greater Central Sensitization Inventory scores associated with worse pain outcomes after endometriosis surgery?

**Findings:**

In this longitudinal presurgery and postsurgery cohort study of 239 people with endometriosis, higher Central Sensitization Inventory scores at baseline were statistically significantly associated with higher chronic pelvic pain scores at follow-up.

**Meaning:**

These findings suggest that the Central Sensitization Inventory may be used preoperatively by gynecologists to counsel patients on expected postoperative pain outcomes and identify people who may have persistent pain after surgery.

## Teaser

This cohort study explores the association between Central Sensitization Inventory scores and persistent pain after endometriosis surgery.

## Introduction

Endometriosis is characterized by the presence of endometrial-like tissue outside the uterine cavity.^1^ It is associated with infertility and pelvic pain, including dysmenorrhea, dyschezia, dyspareunia, and chronic pelvic pain (CPP).^2,3,4,5^ Surgery is a common treatment for endometriosis, including fertility-sparing surgery (eg, excision of lesions) and hysterectomy (with or without oophorectomy, ideally combined with excision).^6^ Potential reasons for proceeding to surgery in this population include persistent pain or adverse effects with hormonal therapy, trying to conceive, or patient choice. However, surgery is not adequate for complete resolution of symptoms in some people.^7,8^ Reoperation rates after conservative fertility-sparing surgery can reach 50% within 5 years, suggesting that pain mechanisms beyond the endometriosis lesions themselves may be involved.^9^ In addition, recurrent pain and reoperation can occur in patients undergoing hysterectomy.^10^ Pain mechanisms in endometriosis are multifactorial.^11^

Of note, central sensitization has been increasingly recognized as a crucial component in the pathogenesis of endometriosis-associated pain, occurring alongside the peripheral nociceptive contributors on a continuum.^11^ It is characterized by long-lasting hyperexcitability of the central nociceptive system that can amplify and perpetuate the perception of pain, even after medical or surgical treatment of the endometriotic tissue.^4,12,13,14,15,16^ An increasing body of evidence for central sensitization in endometriosis involves the use of neurophysiologic testing, such as quantitative sensory testing (QST), and magnetic resonance imaging (MRI). Studies using QST have shown that patients with endometriosis have significantly altered pain thresholds after stimulation of both endometriosis sites and extrapelvic locations, suggesting the amplification of central pain processing.^17,18,19^ Brain MRI studies have identified regional changes in brain morphology and function, such as thalamic gray matter volume reduction and alterations in functional connectivity in endometriosis-associated CPP.^20,21^ Such changes in brain function and morphologic mechanisms may contribute to the development of chronic pain and associated comorbidities, such as mood disorders and cognitive impairment.^20,21^ A limitation of QST and MRI is that they are less feasible for implementation into clinical practice.

The Central Sensitization Inventory (CSI) is a validated self-reported questionnaire that is used to assess symptom severity and identify people with central sensitivity syndromes (CSSs), also referred to as chronic overlapping pain conditions, where central sensitization may be part of the underlying cause.^22,23,24,25^ This questionnaire assesses systemic pain and pain-related symptoms and mental health but is not a neurophysiological measure and thus not a direct marker of central sensitization.^26^ A previous study^27^ found that the number of CSSs was significantly correlated with increasing pain scores and CSI scores in endometriosis. Although the CSI is not a direct or exclusive measure of central sensitization,^26,28,29,30^ it is able to discriminate between patients with CSSs, such as fibromyalgia, and healthy controls.^25,31,32^ The CSI’s measurement properties indicate excellent internal consistency (0.87-0.91), good test-retest reliability (0.82-0.97), and high construct validity (*r* = 0.46 for larger regions of pain).^23,24,25,31,33,34^

Endometriosis costs approximately $16 000 annually per patient in the US,^35^ and at a tertiary center, patients with pelvic pain reported up to 20 operations before referral, with a possible explanation being unrecognized and untreated central sensitization and overreliance on surgery.^36^ Thus, an important clinical problem in the management of endometriosis is the ability to identify individuals who may not benefit from surgery in order to prevent unnecessary additional operations. Currently, there is a lack of clinical tools to counsel patients with endometriosis on their probable pain response to surgery. Therefore, an easy-to-use tool for clinicians to assist them in identifying symptoms that may indirectly reflect central sensitization may help prevent unnecessary interventions for endometriosis.

In this prospective, longitudinal study, the association between CSI and persistent pain after endometriosis surgery was explored, with a primary outcome of CPP and secondary outcomes of dysmenorrhea, deep dyspareunia, dyschezia, and back pain. We hypothesized that higher baseline CSI scores would be associated with worse pain outcomes. In a corollary analysis, we also measured the change in CSI score after surgery to assess the association of surgery with symptoms of central sensitization.

## Methods

### Participants

Prospective, longitudinal data from a patient registry, the Endometriosis Pelvic Pain Interdisciplinary Cohort (EPPIC), were analyzed at a tertiary center for endometriosis and pelvic pain. The EPPIC registry has been described previously.^27,36^ After patients provide consent to EPPIC, the following data sources are entered prospectively and in real time into the registry: (1) patient-reported questionnaires at baseline and follow-up and (2) clinician-reported data for the baseline physical examination and the subsequent index surgery. Research ethics approval for this study was granted by the University of British Columbia and the British Columbia Children’s and Women’s Research ethics boards. All people included in this study consented to their data being used for research as part of the EPPIC data registry; therefore, reconsent was not required for data analysis in this study (approved by the Children's and Women's Research Ethics Board). All data were deidentified. This study followed the Strengthening the Reporting of Observational Studies in Epidemiology (STROBE) reporting guideline for cohort studies.

The study cohort was selected from registry participants who met the following inclusion criteria: (1) 18 to 50 years of age; (2) endometriosis based on a previous surgical diagnosis (visual only or visual and histopathologically confirmed), current endometrioma on imaging or current deep infiltrating nodule on palpation or imaging, or clinically suspected endometriosis; and (3) new or additional referral baseline visit to the center between January 1, 2018, and December 31, 2019, among patients who consented to inclusion in the registry and who prospectively underwent an index surgery (approximately 6 months after baseline visit) by an endometriosis specialist surgeon with advanced training in laparoscopic surgery at our center. We included both conservative (fertility-sparing) surgery, which primarily involved excision of endometriosis lesions, and hysterectomy (with or without bilateral salpingo-oophorectomy, combined with excision of any concurrent lesions). The sample size was too small to divide hysterectomies by oophorectomy status; thus, hysterectomies were considered a single group. Our group aims to excise all visually suspected lesions, both typical and atypical, for all stages (I-IV) and anatomical subtypes (deep, ovarian, and superficial).

**Figure 1.**
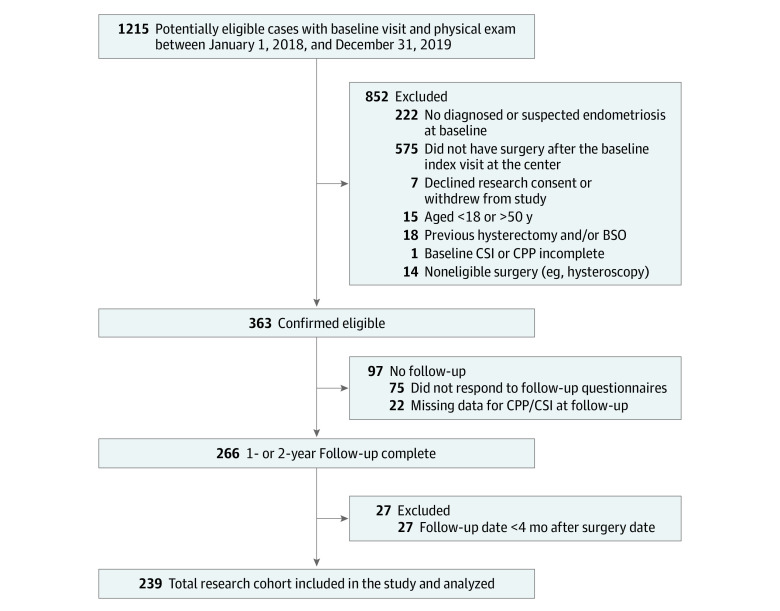
Study Sample Inclusion and exclusion criteria to produce the final study sample. BSO indicates bilateral salpingo-oophorectomy; CPP, chronic pelvic pain; CSI, Central Sensitization Inventory.

The exclusion criteria for participants included (1) postmenopausal status (spontaneous or surgical); (2) previous hysterectomy and/or bilateral salpingo-oophorectomy (before the baseline visit); (3) missing CSI or CPP scores; (4) follow-up completed less than 4 months after surgery; and (5) withdrawn consent. Follow-up questionnaires are sent 1 and 2 years after baseline, although there is variability in when participants actually complete the questionnaires. We used the most recently completed follow-up questionnaire available. Our predetermined goal was to follow up patients through approximately 18 months after surgery. The study cohort is presented in Figure 1.

### Measures

The primary outcome was CPP at follow-up. The secondary outcomes were deep dyspareunia, dysmenorrhea, dyschezia, and back pain. All the pain scores were rated on an 11-point numeric rating scale (0-10, with 0 indicating no pain and 10 indicating worst pain imaginable), and because assumptions of linear regression were not met (normality), we then categorized the pain scores as none to mild (scores, 0-3), moderate (scores, 4-6), and severe (scores, 7-10) for ordinal regression. In the registry, follow-up questionnaires are sent at specific intervals (eg, 1 year and 2 years after baseline), but there is variability when individuals complete the questionnaire: for each participant, we used the most recent follow-up questionnaire available (ie, longest duration of follow-up).

The main variable of interest was the CSI score at baseline measured from 0 to 100, consisting of 25 self-reported questions rated from 0 to 4 (never, rarely, sometimes, often, and always, respectively).^25^ Each of the questions targets a different component of downstream symptoms, such as feeling depressed or having low energy, muscle stiffness, poor sleep, and light sensitivity. A higher score indicates greater symptom severity that may be indirectly related to conditions with underlying central sensitization. The CSI is in the public domain and is freely available.^37^

Other variables considered included demographic characteristics (age, martial status, highest level of education, race, parity, and endometriosis stage), type of surgery (conservative [fertility-sparing] or hysterectomy with or without bilateral salpingo-oophorectomy), and number of CSSs. Patient self-reported race from the EPPIC data registry was included as a variable in this study because there is potential clinical variation by race in endometriosis. As previously reported, we assessed 10 CSSs and pelvic pain-related comorbidities: (1) fibromyalgia, (2) chronic fatigue syndrome, and (3) migraines (each self-reported); (4) irritable bowel syndrome^38,39^ and (5) painful bladder syndrome^40,41^ via diagnostic criteria; (6) bladder or pelvic floor tenderness^42^ and (7) abdominal wall pain^18,36^ via physical examination; (8) anxiety symptoms measured by Generalized Anxiety Disorder 7 (GAD-7)^43^; (9) depressive symptoms measured by Patient Health Questionnaire 9 (PHQ-9)^44^; and (10) magnification, rumination, and helplessness measured by the Pain Catastrophizing Scale.^45^

### Statistical Analysis 

Data analysis was performed from July 2021 to June 2022. The outcomes were examined for associations with baseline CSI scores, as well as with other variables of interest, using ordinal logistic regression and controlling for baseline pain score. Ordinal regression was performed due to failure to meet the normality assumption of linear regression when the 0 to 10 values were used for the primary and secondary outcomes. The assumption of proportional odds was met for all ordinal logistic regression models (full likelihood ratio test *P* > .05 and/or by running separate binomial logistic regressions on cumulative dichotomous-dependent variables). Significant variables on univariable ordinal regressions were put into a multivariable model, followed by backward elimination. Bivariate associations (2-tailed) were performed using an unpaired, independent *t* test or paired-sample *t* test (continuous variables) or a χ^2^ test, Fisher exact test, or Wilcoxon signed rank test (categorical variables). Effect sizes were calculated as follows: (*z* divided by the square root of B) for the Wilcoxon signed rank test and (mean divided by SD) for the paired-sample *t* test Cohen d. SPSS Statistics, version 24 (IBM Corp) was used for analysis. Significance was set at a 2-sided *P* < .05. Missing data were excluded on a per analysis basis.

## Results

### Study Sample

For the 239 included patients (mean [SD] age, 34 [7] years; 189 [79.1%] White [11 (5.8%) identified as White mixed with another race or ethnicity], 1 [0.4%] Black or African American, 29 [12.1%] Asian, 2 [0.8%] Native Hawaiian or Pacific Islander, 16 [6.7%] other, and 2 [0.8%] mixed) with follow-up of more than 4 months after surgery, the mean (SD) baseline CSI score was 43.8 (18.2). The mean (SD) number of days between baseline (ie, completion of CSI) and the index surgery was 227 (143) (range, 14-675) days. For the index surgery, 168 patients (70.3%) underwent conservative surgery, and 71 (29.7%) underwent hysterectomy. A total of 122 (51.0%) had stage I/II endometriosis, whereas 97 (40.6%) had stage III/IV endometriosis. The 239 patients had a mean (SD) follow-up of 16.1 (6.1) months (range, 4.2-27.3 months) after the index surgery, with 97 patients lost to follow-up (leaving 239 of 336 patients for a follow-up rate of 71.0%) (Figure 1). Other sample characteristics are given in eTable 1 in Supplement 1, including other demographic characteristics, characteristics of the index surgery, and other diagnoses.

Those lost to follow-up were more likely to experience baseline moderate back pain (46 [51.7%] vs 80 [37.4%] in the follow-up group; *P* = .04), higher anxiety (mean [SD] GAD-7 score, 8.7 [5.5] vs 6.5 [5.4]; *P* = .001), and depressive symptoms (mean [SD] PHQ-9 score, 10.9 [7.1] vs 8.9 [6.1]; *P* = .02), as well as painful bladder syndrome (58 [59.8%] vs 110 [46.0%]; *P* = .03) and bladder or pelvic floor tenderness (54 [55.7%] vs 95 [39.7%]; *P* = .01) compared with the study cohort (eTable 2 in Supplement 1). There were no other significant differences between the 2 cohorts.

Pain scores significantly decreased from baseline to follow-up after surgery (eTable 3 in Supplement 1), including for the primary outcome of CPP (130 [54.5%] with severe pain at baseline vs 56 [23.4%] with severe pain at follow-up; *P* < .001) and the secondary outcomes of dysmenorrhea (151 [63.2%] with severe pain at baseline vs 54 [22.6%] with severe pain at follow-up; *P* < .001), deep dyspareunia (120 [50.2%] with severe pain at baseline vs 34 [14.2%] with severe pain at follow-up; *P* < .001), dyschezia (99 [41.4%] with severe pain at baseline vs 43 [18.0%] with severe pain at follow-up; *P* < .001), and back pain (111 [46.4%] with severe pain at baseline vs 68 [28.5%] with severe pain at follow-up; *P* < .001) (moderate effect sizes, 0.42-0.60). However, there remained variability in the pain scores at follow-up, with some individuals having persistently high pain (eTable 3 in Supplement 1). When baseline scores were compared with follow-up scores, 131 patients (54.8%) had improved CPP (≤−2 on an 11-point numeric rating scale), 88 (36.8%) stayed the same (no change in pain score or −1 or 1 change), and 20 (8.4%) had pain that worsened (≥2). Therefore, we examined whether baseline CSI scores were associated with greater follow-up pain severity.

### Chronic Pelvic Pain 

Higher baseline CSI scores were significantly associated with higher CPP scores at follow-up (odds ratio [OR], 1.02; 95% CI, 1.00-1.03; *P* = .02), controlling for baseline CPP (Table 1). Of note, although patients with higher CSI scores showed improvement in CPP on average, the degree of improvement was less than for patients with lower CSI scores; that is, patients with higher CSI scores at baseline were more likely to still have persistently elevated pain scores at follow-up (eFigure in Supplement 1).

**Table 1.** Ordinal Regression Between Chronic Pelvic Pain Score at Follow-up, Controlling for Baseline Chronic Pelvic Pain Score

Variable	OR (95% CI)	*P* value
Univariable analyses^a^		
CSI at baseline (continuous)	1.02 (1.00-1.03)	.02
Age	0.94 (0.90-0.97)	<.001
Married (yes vs no)	0.67 (0.39-1.13)	.13
Educational level (at least college vs high school)	0.71 (0.35-1.41)	.32
Stage (III/IV vs I/II)	0.91 (0.53-1.54)	.72
Race (White vs other)^b^	0.87 (0.47-1.62)	.67
Parous (yes vs no)	0.92 (0.54-1.59)	.77
Surgery (hysterectomy vs conservative surgery)	0.39 (0.22-0.70)	.002
Multivariable model with significant variables after backward elimination^c^		
CSI at baseline (continuous)	1.02 (1.00-1.03)	.03
Age, y	0.94 (0.90-0.97)	.001

Abbreviations: CSI, Central Sensitization Inventory; OR, odds ratio.

^a^ Each row is a univariable analysis that is adjusted for baseline pain scores.

^b^ White was compared against all other combined racial groups because of sample size limitations. Most patients (189 [79.1%]) identified as White, of whom 11 (5.8%) identified as White mixed with another race or ethnicity. The other group includes Black or African American (1 [0.4%]), Asian (29 [12.1%]), Native Hawaiian or Pacific Islander (2 [0.8%]), other (16 [6.7%]), and mixed (2 [0.8%]).

^c^ Initial multivariable model with CSI, age, and type of surgery, adjusting for baseline pain scores; after backward elimination, type of surgery fell out of the model. The multivariable regression model results before backward elimination are presented in eTable 7 in Supplement 1.

Older age and hysterectomy (vs conservative surgery) were associated with less severe CPP scores at follow-up. Stage of endometriosis was not significantly associated with CPP at follow-up. In the multivariable model, higher CSI scores at baseline remained significantly associated with a more severe CPP score at follow-up (OR, 1.02; 95% CI, 1.00-1.03; *P* = .03). We repeated the analyses after removing the 8 people with bilateral salpingo-oophorectomy, and the results were similar (eTable 4 in Supplement 1). We also considered the potential impact of the duration of follow-up (surgery to follow-up questionnaire). The duration of follow-up was not associated with CPP at follow-up and did not change the association with CSI (eTable 5 in Supplement 1).

### Other Pain Scores 

We excluded individuals without menses when evaluating dysmenorrhea and those not sexually active when evaluating deep dyspareunia (eTable 3 in Supplement 1). Higher CSI scores at baseline were significantly associated with higher scores for deep dyspareunia (OR, 1.03; 95% CI, 1.01-1.04; *P* = .004), dyschezia (OR, 1.03; 95% CI, 1.01-1.04; *P* < .001), and back pain (OR, 1.02; 95% CI, 1.00-1.03; *P* = .02) at follow-up, controlling for baseline pain scores (Table 2). The associations between higher CSI scores and more severe deep dyspareunia, dyschezia, and back pain at follow-up remained significant in the multivariable regression models (Table 2). In addition, although CSI scores were not significantly associated with dysmenorrhea on the univariable model, CSI was significantly associated in the multivariable model adjusted for surgery group (Table 2). This analysis was followed up with a subanalysis of people who underwent conservative surgery only, and we confirmed that CSI was significantly associated with dysmenorrhea at follow-up (eTable 6 in Supplement 1). See eTable 7 in Supplement 1 for multivariable models before backward elimination.

**Table 2.** Ordinal Regression Between Secondary Pain Outcomes at Follow-up (Ordinal) and Variables of Interest, Controlling for Baseline Pain Scores

Variable	OR (95% CI)	*P* value
**Deep dyspareunia**		
Univariable analyses^a^^,^^b^		
CSI score at baseline (continuous)	1.03 (1.01-1.04)	.004
Age	0.93 (0.88-0.97)	<.001
Married (yes vs no)	0.75 (0.38-1.49)	.42
Educational level (at least college vs high school)	0.35 (0.15-0.79)	.01
Stage (III/IV vs I/II)	0.58 (0.30-1.15)	.12
Race (White vs other)^c^	1.25 (0.57-2.73)	.58
Parous (yes vs no)	0.91 (0.48-1.71)	.76
Surgery (hysterectomy vs conservative surgery)	0.40 (0.20-0.80)	.009
Multivariable model with all significant variables after backward elimination^a^^,^^d^		
CSI score at baseline (continuous)	1.02 (1.00-1.04)	.01
Age	0.94 (0.89-0.98)	.004
Educational level (at least college vs high school)	0.40 (0.17-0.94)	.04
**Dysmenorrhea**		
Univariable analyses^b^^,^^e^		
CSI score at baseline (continuous)	1.01 (1.00-1.03)	.15
Age	0.94 (0.89-0.99)	.02
Married (yes vs no)	0.58 (0.28-1.19)	.14
Educational level (at least college vs high school)	0.45 (0.16-1.22)	.12
Stage (III/IV vs I/II)	0.82 (0.42-1.61)	.57
Race (White vs other)^c^	1.77 (0.81-3.87)	.15
Parous (yes vs no)	1.20 (0.56-2.60)	.64
Surgery (hysterectomy vs conservative surgery)	0.06 (0.01-0.27)	<.001
Multivariable model with all significant variables after backward elimination^d^^,^^e^		
CSI score at baseline (continuous)	1.02 (1.00-1.04)	.03
Surgery (hysterectomy vs conservative surgery)	0.04 (0.01-0.21)	<.001
**Dyschezia**		
Univariable analyses^b^		
CSI score at baseline (continuous)	1.03 (1.01-1.04)	<.001
Age	0.96 (0.92-1.000)	.03
Married (yes vs no)	0.62 (0.36-1.08)	.09
Educational level (at least college vs high school)	0.70 (0.34-1.44)	.33
Stage (III/IV vs I/II)	0.75 (0.43-1.31)	.31
Race (White vs other)^c^	1.59 (0.80-3.18)	.19
Parous (yes vs no)	1.06 (0.60-1.87)	.84
Surgery (hysterectomy vs conservative surgery)	0.44 (0.24-0.82)	.009
Multivariable model with all significant variables after backward elimination^d^		
CSI score at baseline (continuous)	1.03 (1.01-1.04)	.001
Surgery (hysterectomy vs conservative surgery)	0.43 (0.23-0.80)	.007
**Back pain**		
Univariable analyses^b^		
CSI score at baseline (continuous)	1.02 (1.00-1.03)	.02
Age	0.98 (0.95-1.02)	.33
Married (yes vs no)	0.67 (0.39-1.14)	.14
Educational level (at least college vs high school)	0.47 (0.23-0.96)	.04
Stage (III/IV vs I/II)	0.77 (0.45-1.33)	.35
Race (White vs other)^c^	1.86 (0.99-3.52)	.06
Parous (yes vs no)	1.24 (0.72-2.13)	.44
Surgery (hysterectomy vs conservative surgery)	0.56 (0.32-0.98)	.04
Multivariable model with all significant variables after backward elimination^d^		
CSI score at baseline (continuous)	1.02 (1.00-1.03)	.02
Educational level (at least college vs high school)	0.45 (0.22-0.92)	.03

Abbreviations: CSI, Central Sensitization Inventory; OR, odds ratio.

^a^ Excluding those not sexually active ever at baseline and/or not sexually active for the last 3 months at follow-up.

^b^ Each row is a univariable analysis that is adjusted for baseline pain scores.

^c^ White was compared against all other combined racial groups because of sample size limitations. Most patients (189 [79.1%]) identified as White, of whom 11 (5.8%) identified as White mixed with another race or ethnicity. The other group includes Black or African American (1 [0.4%]), Asian (29 [12.1%]), Native Hawaiian or Pacific Islander (2 [0.8%]), other (16 [6.7%]), and mixed (2 [0.8%]).

^d^ Initial multivariable model after backward elimination, adjusting for baseline pain scores. The multivariable regression models before backward elimination are presented in eTable 7 in Supplement 1.

^e^ Excluding those without menses at baseline and/or follow-up (eg, after hysterectomy) (n = 131).

### CSI and CSS

**Figure 2.**
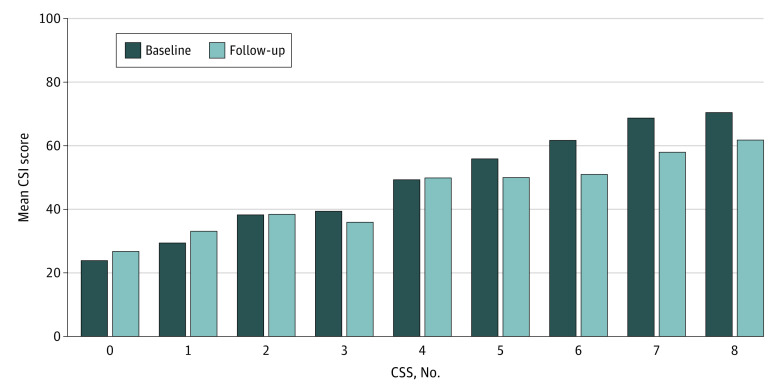
Mean Baseline Central Sensitization Inventory (CSI) Scores at Baseline and Follow-up by Number of Central Sensitivity Syndromes (CSSs) A greater number of CSSs and pelvic pain-related comorbidities was associated with high CSI scores at both baseline (*r* = .71, *P* < .001) and follow-up (*r* = .45, *P* < .001).

There was a statistically significant, but clinically small, improvement in the CSI score at baseline compared with the CSI score at follow-up after surgery (mean [SD], from 43.8 [18.2] to 41.7 [18.9]; effect size, 0.13) (eTable 3 in Supplement 1). We identified a significant association between the number of CSSs and pelvic pain–related comorbidities at baseline and increasing CSI scores at both baseline (*r* = 0.71, *P* < .001) and follow-up (*r* = 0.45, *P* < .001) (Figure 2). As illustrated in Figure 2, CSI scores remained persistently high on average after surgery, particularly in patients with high baseline CSI scores related to a multiple coexistent CSS.

## Discussion

In this prospective study of 239 patients with endometriosis undergoing surgery at a tertiary center, significant reductions of moderate effect size in pain scores were observed on average but with some patients experiencing persistent pain. We found that the baseline CSI score was associated with higher pain scores at follow-up after surgery (both conservative surgery or hysterectomy), including for CPP, deep dyspareunia, dyschezia, and back pain. Furthermore, there was a statistically significant but clinically small reduction in the CSI score itself after surgery; however, CSI scores remained persistently high, especially in those with multiple CSSs.

Our findings suggest that patients with endometriosis with a higher degree of symptom severity (possibly indirectly related to central sensitization) as measured by the CSI at baseline had worse pain outcomes after surgery compared with those with a lesser degree of symptom severity. This finding is in line with our hypothesis because surgical treatment of peripheral pain generators (eg, endometriosis lesions) may not directly treat central nervous system pain generators (eg, central sensitization). Similar to our study, in people who underwent spinal surgery, higher preoperative CSI scores were associated with higher postoperative scores on the Pain Disability Questionnaire.^46^ Furthermore, Roh et al^47^ found that centrally mediated symptoms measured by the CSI were associated with poor functional outcome at 3 months after carpal tunnel surgery, although this association diminished at 12 months. A prospective, observational study by As-Sanie et al^48^ found that the odds of persistent pelvic pain at 6 months after hysterectomy for benign indications increased by 27% for every 1-point increase in central sensitization pain on the Fibromyalgia Survey. Together, these studies^27,46^ indicate that a higher degree of centralized pain may be associated with persistent chronic pain after surgery.

Although pain scores significantly decreased on average with moderate effect size after surgery in our population, there was a statistically significant, but small effect size, change in CSI scores from baseline to follow-up after surgery. This finding is consistent with studies^16,49^ using QST, which found statistically significant but relatively small changes in pain thresholds after endometriosis surgery. Of particular concern were individuals with high CSI scores and multiple CSS comorbidities for whom CSI scores remained persistently high after surgery. These observations suggest that surgery may have little effect on many symptoms of central sensitization, despite improvements in pain. It may be that systemic symptoms of central sensitization, once developed, have a tendency to persist over time despite improvements in other pain-related parameters. However, it remains possible that with longer-term follow-up there may be additional gradual reduction in CSI scores after endometriosis surgery.

### Strengths and Limitations

This study has several strengths, including access to a large patient population from a tertiary center with a 71.0% follow-up rate. This study includes a prospective registry with validated clinical questionnaires (eg, CSI), physical examination, and surgical findings. Endometriosis surgery was also performed by endometriosis specialist surgeons, thereby reducing the possibility of the CPP variable being influenced by significant residual disease. Of note, stage of endometriosis was not associated with the follow-up pain outcomes in our analyses. This finding suggests that inadequate excision and recurrence of disease were likely not factors, even in the high-stage group, because higher pain at follow-up was not found in the people with high-stage disease.

This study also has limitations, including the fact that the CSI mainly measures indirect downstream symptoms, such as pain intensity and sleep disturbances, and that neurophysiological markers of central sensitization were not analyzed. To conclude that the CSI is a useful tool to identify pain sensitization and ultimately alter treatment decisions, the CSI should be correlated with surrogate measures of central sensitization, such as QST, in individuals with endometriosis. In addition, the study setting was a referral center for endometriosis-associated chronic pain, and research is needed to validate the findings in other settings, such as with community gynecologists. This study is also limited by its variability in time between the baseline visit (ie, CSI completion) and index surgery, as well as between index surgery and completion of the follow-up questionnaire (ie, pain outcomes).

## Conclusions

In this cohort study of 239 patients with endometriosis, the CSI at baseline was significantly associated with persistent CPP after endometriosis surgery. These findings suggest that the CSI may be a useful tool to assist clinicians in phenotyping endometriosis-associated pain and in counseling patients on the expected outcomes after surgery.

## Notes

Supplement 1. **eTable 1.** Baseline Characteristics of the Sample (N = 239) **eTable 2.** Follow-Up vs No Follow-Up **eTable 3.** Pain Scores and CSI at Baseline and Post-Surgery Follow-Up (N = 239) **eTable 4.** Ordinal Regression Between Chronic Pelvic Pain Score at Follow-Up, Controlling for Baseline Chronic Pelvic Pain Score (Excluding People With Bilateral Salpingo-Oophorectomy) **eTable 5.** Ordinal Regression Between Chronic Pelvic Pain Score at Follow-up, Controlling for Baseline Chronic Pelvic Pain Score **eTable 6.** Ordinal Regression Between Dysmenorrhea at Follow-Up and Variables of Interest, Controlling for Baseline Pain Scores (Excluding Hysterectomy Group) **eTable 7.** Multivariable Ordinal Regression Models Before Backward Elimination **eFigure.** Chronic Pelvic Pain Scores by Central Sensitization Inventory Group

Supplement 2.
Data Sharing Statement
