# Supplementary material for: Association of Central Sensitization Inventory Scores With Pain Outcomes After Endometriosis Surgery
Source: JAMA Netw Open. 2023 Feb 27;6(2):e230780. doi: 10.1001/jamanetworkopen.2023.0780 (PMC9972194; doi:10.1001/jamanetworkopen.2023.0780)
Supplement: Supplement 1. — eTable 1. Baseline Characteristics of the Sample (N = 239) eTable 2. Follow-Up vs No Follow-Up eTable 3. Pain Scores and CSI at Baseline and Post-Surgery Follow-Up (N = 239) eTable 4. Ordinal Regression Between Chronic Pelvic Pain Score at Follow-Up, Controlling for Baseline Chronic Pelvic Pain Score (Excluding People With Bilateral Salpingo-Oophorectomy) eTable 5. Ordinal Regression Between Chronic Pelvic Pain Score at Follow-up, Controlling for Baseline Chronic Pelvic Pain Score eTable 6. Ordinal Regression Between Dysmenorrhea at Follow-Up and Variables of Interest, Controlling for Baseline Pain Scores (Excluding Hysterectomy Group) eTable 7. Multivariable Ordinal Regression Models Before Backward Elimination eFigure. Chronic Pelvic Pain Scores by Central Sensitization Inventory Group [file jamanetwopen-e230780-s001.pdf]

Supplementary Online Content

Orr NL, Huang AJ, Liu YD, et al. Association of Central Sensitization Inventory scores with pain outcomes after endometriosis surgery. *JAMA Netw Open*. 2023;6(2):e230780. doi:10.1001/jamanetworkopen.2023.0780

eTable 1. Baseline Characteristics of the Sample (N = 239)

eTable 2. Follow-Up vs No Follow-Up

eTable 3. Pain Scores and CSI at Baseline and Post-Surgery Follow-Up (N = 239)

eTable 4. Ordinal Regression Between Chronic Pelvic Pain Score at Follow-Up, Controlling for Baseline Chronic Pelvic Pain Score (Excluding People With Bilateral Salpingo-Oophorectomy)

eTable 5. Ordinal Regression Between Chronic Pelvic Pain Score at Follow-up, Controlling for Baseline Chronic Pelvic Pain Score

eTable 6. Ordinal Regression Between Dysmenorrhea at Follow-Up and Variables of Interest, Controlling for Baseline Pain Scores (Excluding Hysterectomy Group)

eTable 7. Multivariable Ordinal Regression Models Before Backward Elimination

eFigure. Chronic Pelvic Pain Scores by Central Sensitization Inventory Group

This supplementary material has been provided by the authors to give readers additional information about their work.

© 2023 Orr NL et al. *JAMA Network Open.*

eTable 1. Baseline Characteristics of the Sample (N = 239)

Variable	Summary Statistic, No. (%)
Demographics	
**Age, mean±SD, years**	34.12±6.92
**Education**	
College or More (Higher Education)	205 (85.8)
High School or Below	34 (14.2)
**Parity**	
Parous	72 (30.1)
Nulliparous	167 (69.9)
**Partnered (Married or Common-Law)**	
Yes	164 (68.6)
No	75 (31.4)
**Sexual Orientation**	
Heterosexual	206 (86.2)
Lesbian	7 (2.9)
Bisexual	19 (7.9)
None of the above	3 (1.3)
Prefer not to say	4 (1.7)
**Gender Identity**	
Female	236 (98.7)
Non-binary/genderqueer	3 (1.3)
**Currently Working**	
Yes	177 (74.1)
No	45 (18.8)
Missing	17 (7.1)
Endometriosis Diagnosis Before the Index Surgery[a]	
**Previous Histological Diagnosis**	
Yes	62 (25.9)
No	177 (74.1)
**Previous Surgical Visual Diagnosis of Endometriosis**	
Yes	53 (22.2)
No	186 (77.8)
**Endometrioma on Ultrasound at Baseline**	
Yes	56 (23.4)
No	183 (76.6)
**Deep Nodule on Exam or Ultrasound at Baseline**	
Yes	17 (7.1)
No	222 (92.9)

© 2023 Orr NL et al. *JAMA Network Open.*

**Otherwise Clinically Suspected Endometriosis at Baseline**	
Yes	86 (36.0)
No	153 (64.0)
Index Surgery	
**Surgical Approach**	
Laparoscopy	233 (97.5)
Laparotomy	6 (2.5)
**Type of Surgery**	
Conservative	168 (70.3)
Hysterectomy (with/without bilateral salpingo-oophorectomy)	71 (29.7)
With bilateral salpingo-oophorectomy	*8/71(11.3)*
Without bilateral salpingo-oophorectomy	*63/71(88.7)*
**Stage**	
I/II	122 (51.0)
III/IV	97 (40.6)
Missing	20 (8.4)
Endometriosis Identified on Pathology Report	
Superficial Endometriosis	154 (64.4)
Deep Infiltrating Endometriosis	82 (34.3)
Endometrioma	70 (29.3)
Central Sensitivity Syndromes (CSS)	
**CSS total**	
0	17 (7.1)
1	39 (16.3)
2	41 (17.2)
3	46 (19.2)
4	33 (13.8)
5	24 (10.0)
6	17 (7.1)
7	17 (7.1)
8	5 (2.1)
9	0 (0)
10	0 (0)
**Painful Bladder Syndrome**	
Yes	110 (46.0)
No	129 (54.0)
**Irritable Bowel Syndrome**	
Yes	152 (63.6)
No	87 (36.4)
**Abdominal Wall Pain**	
Yes	76 (31.8)
No	163 (68.2)

© 2023 Orr NL et al. *JAMA Network Open.*

**Bladder/Pelvic Floor Tenderness**	
Yes	95 (39.7)
No	144 (60.3)
**PHQ**	
<10	144 (60.3)
>=10	95 (39.7)
**GAD**	
<10	182 (76.2)
>=10	57 (23.8)
**PCS**	
<30	155 (64.9)
>=30	84 (35.1)
**Fibromyalgia**	
Yes	13 (5.4)
No	226 (94.6)
**Chronic Fatigue Syndrome**	
Yes	7 (2.9)
No	232 (97.1)
**Migraines**	
Yes	72 (30.1)
No	167 (69.9)
Co-Treatments in Addition to the Index Surgery	
**Hormone Use (at time of surgery)**	
Yes	120 (50.2)
No	113 (47.3)
Don't know	3 (1.3)
Missing	3 (1.3)
**Hormone Use (at time of follow-up)**	
Yes	101 (42.3)
No	137 (57.3)
Don't know	1 (0.4)
**Counselling**	
Yes	65 (27.2)
No	174 (72.8)
**Physiotherapy**	
Yes	59 (24.7)
No	180 (75.3)
**Pain Education Workshop**	
Yes	60 (25.1)
No	179 (74.9)

Abbreviations: CSS = Central Sensitivity Syndrome; PHQ = Patient Health Questionnaire; GAD = Generalized Anxiety Disorder; PCS = Pain Catastrophizing Scale.

[a]Participants could have more than one type of endometriosis diagnosis before index surgery.

© 2023 Orr NL et al. *JAMA Network Open.*

eTable 2. Follow-Up vs No Follow-Up.

Characteristic	No Follow-Up (N=97)	Follow-Up (N=239)	P Value
	Summary Statistic, No. (%)		
CSI & Pain Scores			
**CSI (0-100) Baseline, mean±SD**	46.73±17.95	43.79±18.15	.18
**CSS Total, mean±SD**	3.66±1.95	3.18±2.06	.05
**CSS Total**			
0	4 (4.1)	18 (7.5)	.06
1	12 (12.4)	39 (16.3)	
2	12 (12.4)	44 (18.4)	
3	21 (21.6)	44 (18.4)	
4	11 (11.3)	31 (13.0)	
5	17 (17.5)	26 (10.9)	
6	15 (15.5)	15 (6.3)	
7	3 (3.1)	18 (7.5)	
8	2 (2.1)	4 (1.7)	
**Fibromyalgia**			
Yes	3 (3.1)	13 (5.4)	.57
No	94 (96.9)	226 (94.6)	
**Chronic Fatigue Syndrome**			
Yes	2 (2.1)	7 (2.9)	>.99
No	95 (97.9)	232 (97.1)	
**Migraines**			
Yes	21(21.6)	72 (30.1)	.14
No	76 (78.4)	167 (69.9)	
**Chronic Pelvic Pain**			
0-3	13 (13.4)	50 (20.9)	.28
4-6	26 (26.8)	59 (24.7)	
7-10	58 (59.8)	130 (54.4)	
**Dysmenorrhea (3 months)**			
0-3	6 (6.6)	27 (12.5)	.14
4-6	23 (25.3)	38 (17.6)	
7-10	62 (68.1)	151 (69.9)	
**Deep Dyspareunia**			
0-3	15 (15.8)	45 (19.6)	.67
4-6	26 (27.4)	65 (28.3)	
7-10	54 (56.8)	120 (52.2)	
**Superficial Dyspareunia**			
0-3	52 (54.7)	146 (62.9)	.09
4-6	28 (29.5)	43 (18.5)	
7-10	15 (15.8)	43 (18.5)	
**Dyschezia**			
0-3	38 (39.2)	90 (37.7)	.74

© 2023 Orr NL et al. *JAMA Network Open.*

4-6	23 (23.7)	50 (20.9)	
7-10	36 (37.1)	99 (41.4)	
**Back Pain**			
0-3	4 (4.5)	23 (10.7)	.04
4-6	46 (51.7)	80 (37.4)	
7-10	39 (43.8)	111 (51.9)	
Demographics			
**Age, , mean±SD, years**	32.98±7.31	34.12±6.92	.18
**Hormone Use (Ever)**			
Yes	84 (91.3)	211 (90.6)	>.99
No	8 (8.7)	22 (9.4)	
**Hormone Use (At time of Surgery)**			
Yes	54 (59.3)	120 (51.5)	.22
No	37 (40.7)	113 (48.5)	
**Ethnicity**			
Caucasian	67 (69.1)	189 (79.1)	.07
Other	30 (30.9)	50 (20.9)	
**Education**			
College or More (Higher Education)	79 (81.4)	205 (85.8)	.32
High School or Below	18 (18.6)	34 (14.2)	
**Parity**			
Parous	37 (38.1)	72 (30.1)	.16
Nulliparous	60 (61.9)	167 (69.9)	
**Partnered (Married or Common-Law)**			
Yes	59 (60.8)	164 (68.6)	.20
No	38 (39.2)	75 (31.4)	
**Sexual Orientation**			
Heterosexual	81 (83.5)	206 (86.2)	.61
Other or Prefer Not to Say	16 (16.5)	33 (13.8)	
**Gender Identity**			
Female	95 (97.9)	236 (98.7)	.63
Non-Binary/Genderqueer/Other	2 (2.1)	3 (1.3)	
**Currently Working**			
Yes	66 (70.2)	177 (79.7)	.08
No	28 (29.8)	45 (20.3)	
**GAD, mean±SD**	8.74±5.49	6.52±5.43	.001
**PHQ, mean±SD**	10.89±7.14	8.88±6.10	.02
**PCS, mean±SD**	26.52±12.55	24.17±12.79	.13
**EHP (total score) , mean±SD**	24.17±12.79	22.69±10.40	.12
**Painful Bladder Syndrome**			
Yes	58 (59.8)	110 (46.0)	.03
No	39 (40.2)	129 (54.0)	
**Irritable Bowel Syndrome**			
Yes	56 (57.7)	152 (63.6)	.32

© 2023 Orr NL et al. *JAMA Network Open.*

No	41 (42.3)	87 (36.3)	
**Abdominal Wall Pain**			
Yes	36 (37.1)	76 (31.8)	.37
No	61 (62.9)	163 (68.2)	
**Bladder/Pelvic Floor Tenderness**			
Yes	54 (55.7)	95 (39.7)	.01
No	43 (44.3)	144 (60.3)	
**Cul-de-Sac Tenderness**			
Yes	58 (59.8)	122 (51.0)	.15
No	39 (40.2)	117 (49.0)	
Endometriosis Diagnosis			
**Suspected Endometriosis**			
Yes	37 (38.1)	86 (36.0)	.71
No	60 (61.9)	153 (64.0)	
**Visual Diagnosis of Endometriosis**			
Yes	23 (23.7)	53 (22.2)	.78
No	74 (76.3)	186 (77.8)	
**Current Endometrioma**			
Yes	23 (23.7)	56 (23.4)	>.99
No	74 (76.3)	183 (76.6)	
**Current Deep Infiltrating Nodule**			
Yes	3 (3.1)	17 (7.1)	.21
No	94 (96.9)	222 (92.9)	
**Previous Histological Diagnosis**			
Yes	24 (24.7)	62 (25.9)	.89
No	73 (75.3)	177 (74.1)	
**Stage**			
I/II	49 (57.0)	121 (55.5)	.90
III/IV	37 (43.0)	97 (44.5)	
Treatment			
**Surgery**			
Hysterectomy group	28 (28.9)	71 (29.7)	>.99
Conservative surgery group	69 (71.1)	168 (70.3)	

Abbreviations: CSI = Central Sensitization Inventory; CSS = Central Sensitivity Syndrome; GAD = Generalized Anxiety Disorder; PHQ = Patient Health Questionnaire; PCS = Pain Catastrophizing Scale; EHP = Endometriosis Health Profile.

© 2023 Orr NL et al. *JAMA Network Open.*

eTable 3. Pain Scores and CSI at Baseline and Post-Surgery Follow-Up (N = 239).

Pain score	Baseline	Follow-up	P Value[a]	Effect Size[f]
	No. (%)			
**Chronic Pelvic Pain**				
0-3	50 (20.9)	120 (50.2)	<.001	.54
4-6	59 (24.7)	63 (26.4)		
7-10	130 (54.4)	56 (23.4)		
**Dysmenorrhea (last 3 Months)**				
0-3	27 (11.3)	51 (21.3)	<.001[b]<br><.001[c]	.51[b]<br>.60[c]
4-6	38 (15.9)	36 (15.1)		
7-10	151 (63.2)	54 (22.6)		
No Menses	23 (9.6)	98 (41.0)		
**Deep Dyspareunia**				
0-3	45 (18.8)	109 (45.6)	<.001[d]	.59[d]
4-6	65 (27.2)	41 (17.2)		
7-10	120 (50.2)	34 (14.2)		
Missing (Not Sexually Active)[e]	9 (3.8)[e]	55 (23.0)[e]		
**Dyschezia**				
0-3	90 (37.7)	146 (61.1)	<.001	.42
4-6	50 (20.9)	50 (20.9)		
7-10	99 (41.4)	43 (18.0)		
**Back Pain**				
0-3	48 (20.1)	106 (44.4)	<.001	.44
4-6	80 (33.5)	65 (27.2)		
7-10	111 (46.4)	68 (28.5)		
**Questionnaire**	**Baseline**	**Follow-up**		
	**Mean ± Standard deviation**			

© 2023 Orr NL et al. *JAMA Network Open.*

**Central Sensitization Inventory (CSI) (0-100)**	43.79±18.15	41.74±18.94	.05	.13

[a]Wilcoxon signed rank test for categorical pain variables; Paired sample t-test for CSI

[b]Excluding those with no menses

[c]Classifying those with no menses as 0-3 score

[d]Excluding those not sexually active

[e]Baseline question is about not sexually active ever; follow-up question is not sexually active last 3 months

[f]Wilcoxon signed rank effect size = z/sqrt[B]; Paired-sample t-test cohen d = mean/SD

© 2023 Orr NL et al. *JAMA Network Open.*

eTable 4. Ordinal Regression Between Chronic Pelvic Pain Score at Follow-Up, Controlling for Baseline Chronic Pelvic Pain Score (Excluding People With Bilateral Salpingo-Oophorectomy).

Variable	P Value	Odds Ratio	95% CI	
			Lower	Upper
**Univariable Analyses[a]**				
CSI at Baseline (continuous)	.02	1.018	1.003	1.033
Age (years)	.001	.938	.903	.975
Married (Yes vs. No)	.22	.716	.418	1.226
Education (At Least College vs. High School)	.33	.704	.349	1.420
Stage (III/IV vs. I/II)	.90	.964	.562	1.655
Ethnicity (Caucasian vs. Other)	.48	.795	.424	1.493
Parous (Yes vs. No)	.90	1.038	.595	1.811
Surgery (Hysterectomy vs. Conservative Surgery)	.004	.420	.232	.761
**Multivariable Model With Significant Variables Before Backwards Elimination**				
CSI at Baseline (continuous)	.02	1.018	1.003	1.033
Age	.04	.956	.916	.998
Surgery (Hysterectomy vs. Conservative surgery)	.08	.550	.282	1.071
**Multivariable Model With Significant Variables After Backwards Elimination**				
CSI at Baseline (continuous)	.03	1.017	1.002	1.032
Age (years)	.001	.940	.904	.976

[a]Each row is an univariable analysis that is adjusted for baseline pain scores.

© 2023 Orr NL et al. *JAMA Network Open.*

eTable 5. Ordinal Regression Between Chronic Pelvic Pain Score at Follow-up, Controlling for Baseline Chronic Pelvic Pain Score.

Variable	P value	Odds Ratio	95% CI Lower	95% CI Upper
**Univariable Model**				
Time (between surgery and follow-up)	.374	.981	.941	1.023
**Multivariable Model**				
Time (between surgery and follow-up)	.392	.982	.940	1.024
CSI at Baseline	.019	1.018	1.003	1.033
Age	.044	.957	.918	.999
Hysterectomy (vs conservative surgery)	.047	.514	.267	.991

© 2023 Orr NL et al. *JAMA Network Open.*

eTable 6. Ordinal Regression Between Dysmenorrhea at Follow-Up and Variables of Interest, Controlling for Baseline Pain Scores (Excluding Hysterectomy Group).

Variable	P Value	Odds Ratio	95% CI	
			Lower	Upper
**Dysmenorrhea[a] – Univariable Analyses[b]**				
CSI at Baseline (continuous)	.05	1.020	1.000	1.040
Age (years)	.44	.977	.922	1.036
Married (Yes vs. No)	.46	.754	.354	1.604
Education (At Least College vs. High School)	.42	.653	.234	1.820
Stage (III/IV vs. I/II)	.48	.773	.376	1.590
Ethnicity (Caucasian vs. Other)	.18	1.760	.767	4.036
Parous (Yes/No)	.50	1.338	.572	3.134

[a]Excluding those without menses at baseline and/or follow-up (e.g., after hysterectomy); n=116

[b]Each row is an univariable analysis that is adjusted for baseline pain scores.

© 2023 Orr NL et al. *JAMA Network Open.*

eTable 7. Multivariable Ordinal Regression Models Before Backward Elimination.

Variable	P Value	Odds Ratio	Lower 95% CI	Upper 95% CI
**Chronic Pelvic Pain**				
CSI at Baseline (continuous)	.02	1.018	1.003	1.033
Age	.04	.955	.916	.997
Hysterectomy (vs. conservative surgery)	.06	.528	.274	1.015
**Deep Dyspareunia**				
CSI at Baseline (continuous)	.008	1.025	1.007	1.045
Age	.07	.954	.906	1.003
Higher Education (At Least College)	.04	.400	.170	.940
Hysterectomy (vs. conservative surgery)	.11	.523	.239	1.146
**Dysmenorrhea**				
CSI at Baseline (continuous)	.03	1.022	1.002	1.042
Age	.61	.985	.929	1.045
Hysterectomy (vs. conservative surgery)	.001	.051	.009	.269
**Dyschezia**				
CSI at Baseline (continuous)	.001	1.028	1.011	1.044
Age	.53	.986	.944	1.030
Hysterectomy (vs. conservative surgery)	.04	.474	.236	.949
**Back Pain**				
CSI at Baseline (continuous)	.02	1.019	1.003	1.034
Higher Education (At Least College)	.05	.482	.233	.999
Hysterectomy (vs. conservative surgery)	.07	.592	.337	1.041

© 2023 Orr NL et al. *JAMA Network Open.*

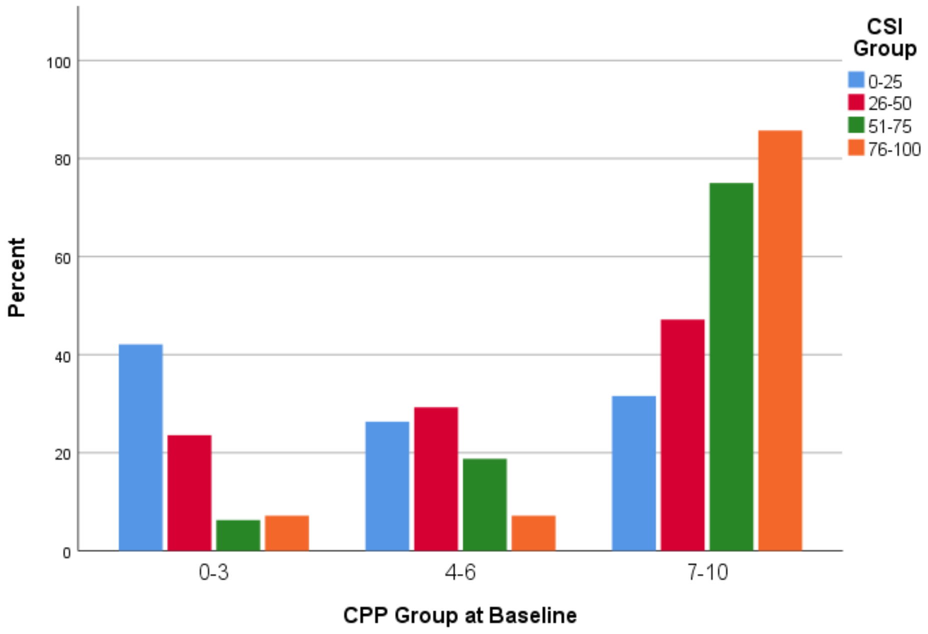

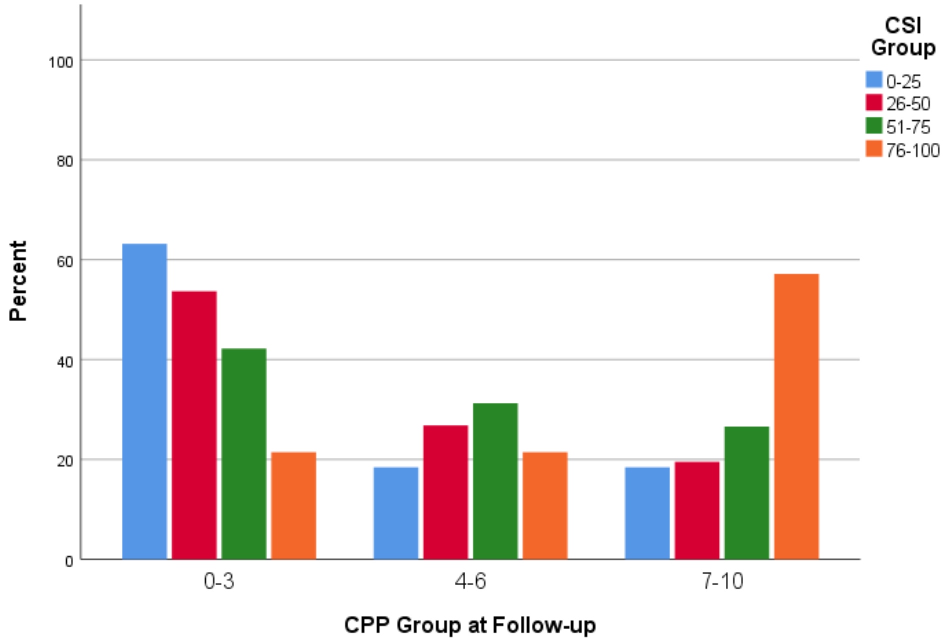

eFigure 1. Chronic Pelvic Pain Scores by Central Sensitization Inventory Group.

The top figure represents the chronic pelvic pain (CPP) scores at baseline and the bottom figure represents the CPP scores at follow-up. The Central Sensitization Inventory (CSI) scores at baseline were divided into four groups and the percentage of people within each CPP group per CSI group was shown.

© 2023 Orr NL et al. *JAMA Network Open.*
