# Supplementary material for: Association of Central Sensitization Inventory Scores With Pain Outcomes After Endometriosis Surgery
Source: JAMA Netw Open. 2023 Feb 27;6(2):e230780. doi: 10.1001/jamanetworkopen.2023.0780 (PMC9972194; doi:10.1001/jamanetworkopen.2023.0780)
Supplement: Supplement 2. — Data Sharing Statement [file jamanetwopen-e230780-s002.pdf]

Data Sharing Statement

Orr. Association of Central Sensitization Inventory Scores With Pain Outcomes After Endometriosis Surgery. *JAMA Netw Open*. Published February 27, 2023. doi:10.1001/jamanetworkopen.2023.0780

Data
Data available: No

Additional Information
Explanation for why data not available: We do not have REB approval for data sharing from the registry
